# The association between body mass index and osteoporosis in a Taiwanese population: a cross-sectional and longitudinal study

**DOI:** 10.1038/s41598-024-59159-4

**Published:** 2024-04-12

**Authors:** Chao-Tse Chiu, Jia-In Lee, Cheng-Chang Lu, Shu-Pin Huang, Szu-Chia Chen, Jiun-Hung Geng

**Affiliations:** 1 Department of Orthopaedics, Kaohsiung Municipal Siaogang Hospital, Kaohsiung, Taiwan; 2 Department of Orthopaedics, Kaohsiung Medical University Hospital, Kaohsiung Medical University, Kaohsiung, Taiwan; 3 Department of Psychiatry, Kaohsiung Medical University Hospital, Kaohsiung Medical University, Kaohsiung, Taiwan; 4 Department of Urology, Kaohsiung Medical University Hospital, Kaohsiung Medical University, Kaohsiung, 807 Taiwan; 5 Department of Urology, School of Medicine, College of Medicine, Kaohsiung Medical University, 807 Kaohsiung, Taiwan; 6 Graduate Institute of Clinical Medicine, College of Medicine, Kaohsiung Medical University, Kaohsiung, 807 Taiwan; 7 Research Center for Environmental Medicine, Kaohsiung Medical University, Kaohsiung, 807 Taiwan; 8 Ph.D. Program in Environmental and Occupational Medicine, College of Medicine, Kaohsiung Medical University, Kaohsiung, 807 Taiwan; 9 Institute of Medical Science and Technology, College of Medicine, National Sun Yat-Sen University, Kaohsiung, 804 Taiwan; 10 Department of Internal Medicine, Kaohsiung Municipal Siaogang Hospita, l, Kaohsiung Medical University, 812 Kaohsiung, Taiwan; 11 Division of Nephrology, Department of Internal Medicine, Kaohsiung Medical University Hospital, Kaohsiung Medical University, Kaohsiung, 807 Taiwan; 12 Faculty of Medicine, College of Medicine, Kaohsiung Medical University, Kaohsiung, 807 Taiwan; 13 Department of Urology, Kaohsiung Municipal Siaogang Hospital, No. 482, Shanming Rd, Xiaogang District, Kaohsiung, 812 Taiwan

**Keywords:** Epidemiologic study, Osteoporosis, Body mass index, Taiwan Biobank, Bone mineral density, Risk factors, Obesity, Malnutrition

## Abstract

This study investigates the correlation between body mass index (BMI) and osteoporosis utilizing data from the Taiwan Biobank. Initially, a comprehensive analysis of 119,009 participants enrolled from 2008 to 2019 was conducted to assess the association between BMI and osteoporosis prevalence. Subsequently, a longitudinal cohort of 24,507 participants, initially free from osteoporosis, underwent regular follow-ups every 2–4 years to analyze the risk of osteoporosis development, which was a subset of the main cohort. Participants were categorized into four BMI groups: underweight (BMI < 18.5 kg/m^2^), normal weight (18.5 kg/m^2^ ≤ BMI < 24 kg/m^2^), overweight (24 kg/m^2^ ≤ BMI < 27 kg/m^2^), and obese groups (BMI ≥ 27 kg/m^2^). A T-score ≤ − 2.5 standard deviations below that of a young adult was defined as osteoporosis. Overall, 556 (14.1%), 5332 (9.1%), 2600 (8.1%) and 1620 (6.7%) of the participants in the underweight, normal weight, overweight and obese groups, respectively, had osteoporosis. A higher prevalence of osteoporosis was noted in the underweight group compared with the normal weight group (odds ratio [OR], 2.20; 95% confidence interval [95% CI], 1.99 to 2.43; p value < 0.001) in multivariable binary logistic regression analysis. Furthermore, in the longitudinal cohort during a mean follow-up of 47 months, incident osteoporosis was found in 61 (9%), 881 (7.2%), 401 (5.8%) and 213 (4.6%) participants in the underweight, normal weight, overweight and obese groups, respectively. Multivariable Cox proportional hazards analysis revealed that the risk of incident osteoporosis was higher in the underweight group than in the normal weight group (hazard ratio [HR], 1.63; 95% CI 1.26 to 2.12; p value < 0.001). Our results suggest that BMI is associated with both the prevalence and the incidence of osteoporosis. In addition, underweight is an independent risk factor for developing osteoporosis. These findings highlight the importance of maintaining normal weight for optimal bone health.

## Introduction

Osteoporosis, a pervasive bone metabolic disorder characterized by diminished bone mineral density (BMD) and compromised bone microarchitecture, poses a formidable challenge to global public health^1^. The disorder results in skeletal fragility and significantly elevates the risk of fractures and consequently the burden of morbidity and mortality, particularly concerning hip fractures in older age^2^. The determinants of osteoporosis are multifaceted and can be categorized into two principal domains: nonmodifiable and modifiable factors. Nonmodifiable factors encompass genetic variations, advanced age, ethnicity, sex, reproductive status, and chronic estrogen deprivation, and underlie the complexity of the etiology of osteoporosis^3^. Of these factors, advanced age and female sex are the most prominent and important risk factors for osteoporosis^4^.

In contrast to the nonmodifiable factors, modifiable factors present intriguing avenues for interventions and prevention, and include dietary habits^5^, sedentary lifestyles^6^, body composition^7^, body weight^7^, smoking^8^, prolonged corticosteroid therapy^9^, excessive alcohol^10^, coffee consumption^11^, and Vitamin D deficiency^12^. Among these factors, the interaction between body weight and bone mass plays a crucial role in bone health^7^. Mechanical loading due to body weight is an important element of this relationship, and underscores the importance of weight-bearing exercises and activities. Previous research has provided compelling evidence demonstrating that obesity and weight gain are positively correlated with higher BMD and less bone loss^13,14^. Conversely, thinness and weight loss are associated with lower BMD and an higher rate of bone loss, further emphasizing the critical role of body weight in skeletal health^13,14^.

Despite advances in our understanding of osteoporosis^14,15^, a significant research gap remains, particularly in the context of large-scale epidemiological studies in Asian populations. Specifically, the relationship between body mass index (BMI) and osteoporosis in Asian populations remains insufficiently explored. To address this knowledge gap, the aim of this study was to examine the nuanced connections between BMI and osteoporosis using a comprehensive dataset provided by the Taiwan Biobank (TWB), a robust population-based repository.

## Materials and methods

### Study participants and ethics statement

**Figure 1:**
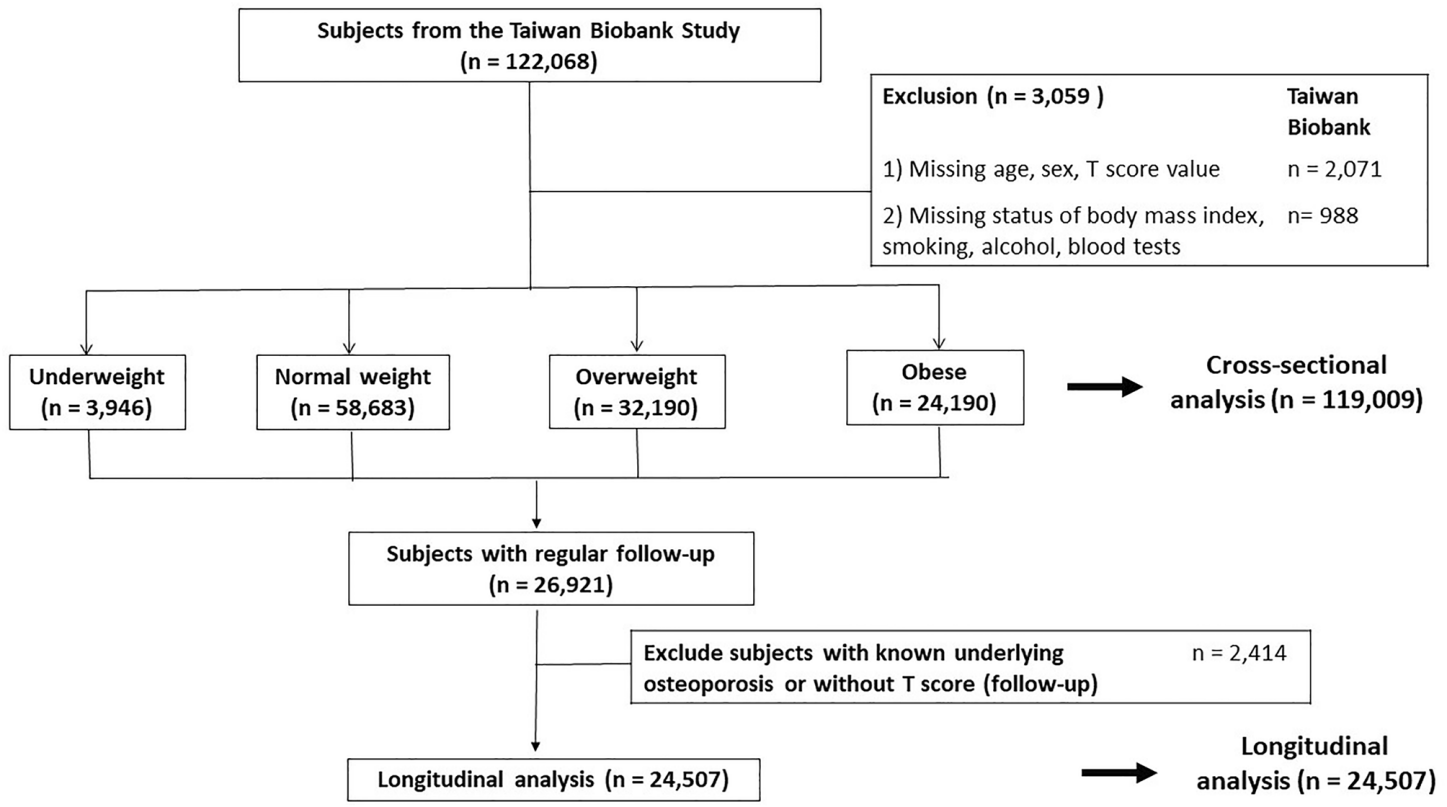
Study participants were classified by body mass index.

We recruited participants from the TWB dataset, a comprehensive population-based biobank in Taiwan, which aimed at collecting and storing biological samples, along with associated health and lifestyle data, from a diverse population in Taiwan. It serves as a valuable resource for researchers studying various aspects of health and disease, including genetics, environmental influences, and lifestyle factors. The biobank facilitates investigations into the relationships between environmental exposures, and the development of diseases, with the ultimate goal of advancing medical research, improving healthcare outcomes, and facilitating personalized medicine initiatives. The objectives, methodology, and detailed information of the TWB have been documented previously^16–19^. BMI and BMD data were available for most of the participants, enabling us to investigate the relationship between BMI and osteoporosis. Initially, a cohort of 122,068 participants enrolled in the TWB from 2008 to 2019, as illustrated in Fig. 1. After excluding individuals with missing data (n = 3059), the final analysis encompassed 119,009 subjects.

Subsequently, a longitudinal cohort of 24,507 participants, initially free from osteoporosis, underwent regular follow-ups every 2–4 years to analyze the risk of osteoporosis development, which was a subset of the main cohort (Fig. 1). As shown in Fig. 1, within the TWB database, 26,921 participants underwent routine follow-up examinations, with those initially diagnosed with osteoporosis (n = 2414) excluded, resulting in 24,507 individuals included in the final analysis. Spanning from 2008 to 2019, these participants underwent regular follow-ups, completed periodic questionnaires, and underwent BMD assessments every 2–4 years.

This study received ethical approval from the Institutional Review Board of Kaohsiung Medical University Hospital (KMUHIRB-E(I)-20210058). All researchers adhered to the principles outlined in the Declaration of Helsinki, and written informed consent was obtained from every participant.

### BMI categories

BMI categories for adults in this study adhere to the WHO classification, which categorizes individuals based on their BMI as follows^20^:

- BMI < 18.5 kg/m^2^: underweight;
- 18.5 kg/m^2^ ≤ BMI < 24 kg/m^2^: normal weight;
- 24 kg/m^2^ ≤ BMI < 27 kg/m^2^: overweight;
- BMI ≥ 27 kg/m^2^: obese.

### Study outcome and definition of osteoporosis

In the cross-sectional analysis, we scrutinized the relationship between BMI and the prevalence of osteoporosis. In the longitudinal analysis, the primary end point was the development of osteoporosis. The definition of osteoporosis in this study involved measuring estimated BMD (g/cm^2^) through calcaneus quantitative ultrasound (QUS) (Achilles InSight, GE, USA). The T-score was calculated using the formula: the individual’s BMD minus the mean BMD in young adults, divided by the standard deviation (SD) of a normal young-adult population^21^. Osteoporosis was diagnosed when the T-score was ≤ − 2.5 SD below that of young adults.

### Variables

Through an extensive literature review, we identified key variables linked to the development of osteoporosis. These variables were age^3^, sex^4^, smoking habits^8^, alcohol consumption^10^, hypertension^22^, diabetes mellitus^23^, dyslipidemia^24^, anemia^25^, serum albumin level^26^, uric acid level^27^, and kidney function^28^. Demographic and lifestyle factors including age, sex, smoking status (classified as participants who had ever smoked vs. those who had never smoked), and alcohol consumption (categorized as participants who consumed > 150c.c/week for 6 months vs. non-consumers) were assessed through self-administered questionnaires. Medical history variables such as past hypertension, diabetes mellitus, and dyslipidemia were also obtained from participant responses. Interviewers were instructed to reiterate these inquiries and verify the consistency of the responses. Clinical parameters including anemia (defined as hemoglobin < 13.5 g/dl for males and < 12.5 g/dl for females), serum albumin levels, uric acid levels, and kidney function (determined through estimated glomerular filtration rate, eGFR) were evaluated through blood tests. eGFR was calculated using the Modification of Diet in Renal Disease (MDRD) formula: eGFR (mL/min/1.73 m^2^) = 175 × (Serum creatinine)^− 1.154^ × (Age)^− 0.203^ × (0.742 if female)^29^.

In the longitudinal cohort study, participants underwent regular follow-ups, typically occurring within 2–4 years following the initial appointment. These follow-ups involved completing periodic questionnaires, undergoing physical examinations, providing blood samples, and undergoing BMD tests.

To ensure the effectiveness and acuracy of data, the TWB has been complemented by the development of its infrastructure. This includes obtaining international organization for standardization (ISO) certification, implementing integrated multi-center recruitment, synthesizing data/information systems, and ensuring international accessibility. Detailed information can be found in previous studies^30,31^.

### Statistical analyses

The participants were stratified into four groups based on their BMI: underweight, normal weight, overweight, and obese. Categorical variables were represented as percentages, while continuous variables were presented as mean ± SD. One-way ANOVA was employed to compare these groups regarding continuous variables, and the Pearson χ^2^ test was used for categorical variables. To assess the association between BMI and osteoporosis prevalence, multivariable binary logistic regression analyses were conducted in the cross-sectional analysis. In addition, to evaluate the association between BMI and the risk of osteoporosis development, Kaplan–Meier and Cox proportional hazard regression analyses were performed in the longitudinal cohort. The Kaplan–Meier analysis, along with a log-rank test, was employed to estimate the cumulative incidence of osteoporosis. Survival time was defined as the duration from baseline assessment to the occurrence of incident osteoporosis or until the last follow-up visit for participants who remained osteoporosis-free. Censorship was applied to individuals lost to follow-up or deceased, with their data being censored at the date of their last examination. Cox proportional hazards analyses were conducted to ascertain the independent association between BMI and the development of osteoporosis. Furthermore, to investigate the correlation between BMI and changes in BMD T-score, one-way ANOVA was applied. Statistical significance was established at p value < 0.05. All statistical analyses were performed using R version 3.6.2 (R Foundation for Statistical Computing, Vienna, Austria) and SPSS version 20.0 (IBM Corp, Armonk, NY, USA).

## Results

### Baseline characteristics of the participant classified by BMI

For the cross-sectional analysis, 119,009 participants were enrolled, with a mean age of 50 ± 11 years (Table 1). Overall, 3% (n = 3946) of the participants were underweight, 49% (n = 58,683) were normal weight, 27% (n = 32,190) were overweight, and 21% (n = 24,190) were obese. Notably, individuals in the underweight group were generally younger, predominantly female, and had lower rates of smoking and drinking compared to the other groups. Moreover, individuals in the underweight group had decreased prevalence rates of hypertension, diabetes mellitus, and dyslipidemia, and also lower levels of serum hemoglobin, fasting glucose, triglycerides, total cholesterol, and uric acid, along with higher BMD T-scores compared to the other groups (Table 1).

**Table 1:** Baseline characteristics of participants in the Taiwan Biobank for the cross-sectional analysis classified by BMI (n = 119,009).

Characteristics	Total	Underweight (BMI < 18.5)	Normal weight (18.5 ≤ BMI < 24)	Overweight (24 ≤ BMI < 27)	Obese (BMI ≥ 27)	p value
Number (%)	119,009	3946 (3)	58,683 (49)	32,190 (27)	24,190 (21)	-
Demographic data						
Age, yr	50 ± 11	46 ± 12	50 ± 11	51 ± 11	49 ± 11	< 0.001
Women, n (%)	76,293 (64)	3392 (86)	43,451 (74)	17,163 (53)	12,287 (51)	< 0.001
Smoking, ever, n (%)	32,431 (27)	652 (17)	12,464 (21)	10,480 (33)	8835 (37)	< 0.001
Alcohol status, ever, n (%)	10,138 (9)	175 (4)	3727 (6)	3388 (11)	2848 (12)	< 0.001
Comorbidities						
Hypertension, n (%)	14,586 (12)	82 (2)	3944 (7)	4978 (16)	5582 (23)	< 0.001
Diabetes, n (%)	6118 (5)	73 (2)	1861 (3)	1941 (6)	2243 (9)	< 0.001
Dyslipidemia, n (%)	8877 (8)	112 (3)	3109 (5)	2879 (9)	2777 (12)	< 0.001
Laboratory data						
Hemoglobin, g/dl	13.8 ± 1.6	13.1 ± 1.4	13.4 ± 1.5	14.0 ± 1.6	14.3 ± 1.6	< 0.001
Albumin, g/dl	4.5 ± 0.2	4.5 ± 0.2	4.5 ± 0.2	4.5 ± 0.2	4.5 ± 0.2	< 0.001
Fasting glucose, mg/dl	96 ± 21	89 ± 14	93 ± 16	98 ± 22	101 ± 27	< 0.001
Total cholesterol, mg/dl	196 ± 36	188 ± 34	195 ± 36	198 ± 36	197 ± 37	< 0.001
Triglyceride, mg/dl	115 ± 94	69 ± 38	95 ± 71	130 ± 100	155 ± 120	< 0.001
Uric acid, mg/dL	5.4 ± 1.4	4.4 ± 1.0	5.0 ± 1.2	5.8 ± 1.4	6.2 ± 1.5	< 0.001
eGFR, ml/min per 1.73 m^2^	103 ± 24	111 ± 25	106 ± 24	100 ± 23	100 ± 24	< 0.001
BMD T score	− 0.387 ± 1.627	− 0.663 ± 1.692	− 0.415 ± 1.643	− 0.417 ± 1.583	− 0.234 ± 1.623	< 0.001

BMI: body mass index; BMD: bone mineral density; eGFR: estimated glomerular filtration rate. Unit of BMI: kg/m^2^.

### Association between BMI and prevalent osteoporosis

Among the 119,009 participants in the cross-sectional analysis, a total of 10,108 individuals (8.5%) were diagnosed with osteoporosis. The prevalence of osteoporosis varied among the BMI groups, with 556 (14.1%) in the underweight group, 5332 (9.1%) in the normal weight group, 2600 (8.1%) in the overweight group, and 1620 (6.7%) in the obese group (Table 2). Univariable binary logistic analysis identified several factors associated with prevalent osteoporosis, including BMI, age, sex, smoking, drinking, history of hypertension, diabetes mellitus, dyslipidemia, serum albumin, fasting glucose, total cholesterol, triglyceride levels, and estimated glomerular filtration rate (Supplementary Table S1). Multivariable binary logistic analyses, adjusting for factors associated with prevalent osteoporosis identified in univariable analyses, revealed that the individuals in the underweight group had a significantly higher odds of osteoporosis compared to those in the normal weight group (odds ratio [OR], 2.20; 95% confidence interval [95% CI], 1.99 to 2.43; p value < 0.001) (Table 2). Conversely, the overweight and obese groups had lower odds of osteoporosis compared to the normal weight group (OR, 0.74; 95% CI 0.71 to 0.78]; p value < 0.001; and OR, 0.69; 95% CI 0.65 to 0.74; p < 0.001, respectively) (Table 2). These findings remained consistent in subgroup analyses stratified by age and sex (Supplementary Table S2).

**Table 2:** Univariable and multivariable binary logistic analyses for the prevalence of osteoporosis in different BMI groups (n = 119,009).

BMI groups	No. of prevalent osteoporosis cases/no. of subjects (%)	Unadjusted odds ratio (95% CI)	p value	Adjusted odds ratio (95% CI)	p value
Underweight (BMI < 18.5)	556/3946 (14.1)	1.64 (1.49 to 1.80)	< 0.001	2.20 (1.99 to 2.43)	< 0.001
Normal weight (18.5 ≤ BMI < 24)	5332/58,683 (9.1)	1.00 (reference)	–	1.00 (reference)	–
Overweight (24 ≤ BMI < 27)	2600/32,190 (8.1)	0.88 (0.84 to 0.92)	< 0.001	0.74 (0.71 to 0.78)	< 0.001
Obese (BMI ≥ 27)	1620/24,190 (6.7)	0.72 (0.68 to 0.76)	< 0.001	0.69 (0.65 to 0.74)	< 0.001

CI: confidence interval. Multivariable model: adjustment for age, sex, smoking, drinking, history of hypertension, history of diabetes mellitus, history of dyslipidemia, serum albumin, fasting glucose, total cholesterol, triglyceride, and estimated glomerular filtration rate.

Unit of BMI = kg/m^2^.

### Association of BMI with the development of osteoporosis

**Figure 2:**
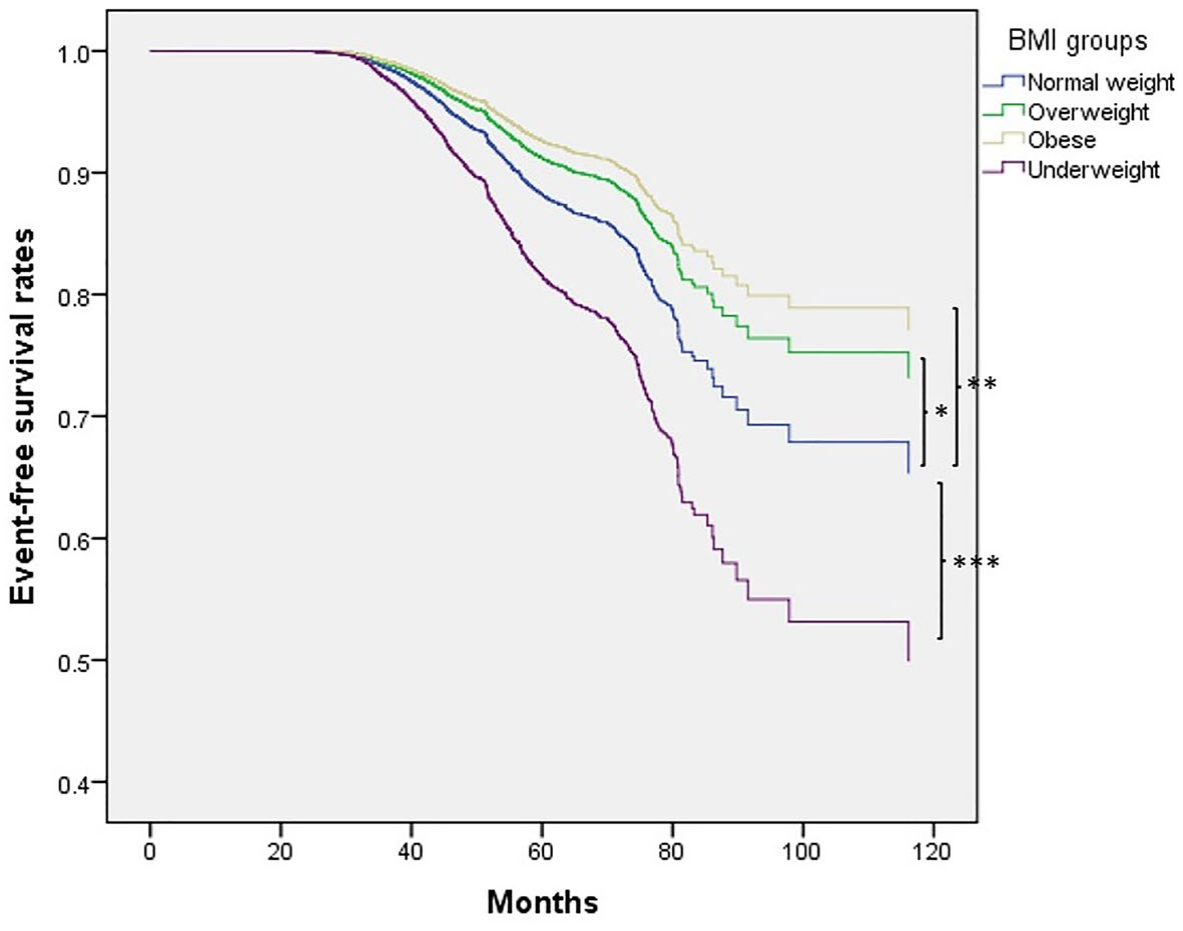
Time to osteoporosis development was shorter in underweight participants than normal weight participants. Kaplan–Meier plot of incident osteoporosis development according to BMI groups in 24,507 participants with follow-up data. *p value < 0.001; **p value < 0.001; ***p value < 0.001.

To further corroborate our findings within a longitudinal cohort, we enrolled 24,507 participants who lacked osteoporosis at baseline to investigate the influence of BMI on osteoporosis development. Participants were stratified into underweight (662, 3%), normal weight (12,321, 50%), overweight (6941, 28%), and obese (4583, 19%) categories (Table 3). During a mean follow-up period of 47 months, newly developed osteoporosis was observed in 1556 (6.3%) of all participants, 61 (9.2%) of the underweight individuals, 881 (7.2%) of the normal weight individuals, 401 (5.8%) of the overweight individuals, and 213 (4.6%) of the obese individuals (Table 4). Univariable analysis revealed associations between BMI, age, sex, history of hypertension, diabetes mellitus, dyslipidemia, serum albumin, fasting glucose, and total cholesterol with incident osteoporosis (Supplementary Table S3). Multivariable Cox proportional hazards regression analysis, adjusting for factors associated with incident osteoporosis as indicated in Supplementary Table S3, demonstrated that the risk of developing incident osteoporosis was greater in the underweight group compared to the normal weight group (hazard ratio [HR], 1.63; 95% CI 1.26 to 2.12; p value < 0.001) (Table 4). Conversely, the overweight and obese groups exhibited a lower risk of developing osteoporosis compared to the normal weight group (Table 4). Kaplan–Meier plots demonstrated that the time to incident osteoporosis development was significantly shorter for participants in the underweight group compared to the normal weight group (Fig. 2).

**Table 3:** Clinical characteristics of the longitudinal cohort classified by BMI (n = 24,507).

Characteristics	Total	Underweight (BMI < 18.5)	Normal weight (18.5 ≤ BMI < 24)	Overweight (24 ≤ BMI < 27)	Obese (BMI ≥ 27)	p value
Number (%)	24,507	662 (3)	12,321(50)	6941 (28)	4583(19)	–
Demographic data						
Age, yr	51 ± 10	46 ± 11	50 ± 10	52 ± 10	51 ± 10	< 0.001
Women	15,977(65)	576 (87)	9125 (74)	3782 (55)	2494 (54)	< 0.001
Smoking, ever	5869 (24)	85 (13)	2224 (18)	2096 (30)	1464 (32)	< 0.001
Alcohol status, ever	2035 (8)	23 (4)	727 (6)	742 (11)	543 (12)	< 0.001
Comorbidities						
Hypertension	3115 (13)	12 (2)	866 (7)	1135 (16)	1102 (24)	< 0.001
Diabetes	1277 (5)	12 (2)	409 (3)	407 (6)	449 (10)	< 0.001
Dyslipidemia	1805 (7)	18 (3)	644 (5)	595 (9)	548 (12)	< 0.001
Laboratory data						
Hemoglobin, g/dl	13.7 ± 1.6	13.1 ± 1.4	13.4 ± 1.5	14.0 ± 1.5	14.1 ± 1.6	< 0.001
Albumin, g/dl	4.6 ± 0.2	4.6 ± 0.2	4.5 ± 0.2	4.6 ± 0.2	4.6 ± 0.2	< 0.001
Fasting glucose, mg/dl	96 ± 20	88 ± 7	93 ± 16	98 ± 21	102 ± 27	< 0.001
Total cholesterol, mg/dl	195 ± 35	184 ± 33	193 ± 35	198 ± 36	197 ± 36	< 0.001
Triglyceride, mg/dl	114 ± 84	68 ± 30	95 ± 63	127 ± 92	151 ± 104	< 0.001
Uric acid, mg/dL	5.5 ± 1.4	4.4 ± 1.0	5.0 ± 1.2	5.8 ± 1.4	6.3 ± 1.5	< 0.001
eGFR, ml/min per 1.73 m^2^	103 ± 24	112 ± 24	105 ± 23	100 ± 23	100 ± 25	< 0.001
Follow-up, months	47 ± 14	47 ± 13	47 ± 14	46 ± 14	47 ± 14	0.124

BMI: body mass index; BMD: bone mineral density; eGFR: estimated glomerular filtration rate. Unit of BMI = kg/m^2^.

**Table 4:** Risk of incident osteoporosis for longitudinal cohort in different BMI groups (n = 24,507).

BMI groups	No. of incident osteoporosis cases/no. of subjects (%)	Multivariable model, HR (95% CI)	p value
Underweight (BMI < 18.5)	61/662 (9.2)	1.63 (1.26 to 2.12)	< 0.001
Normal weight (18.5 ≤ BMI < 24)	881/12,321 (7.2)	1.00 (reference)	–
Overweight (24 ≤ BMI < 27)	401/6941 (5.8)	0.73 (0.65 to 0.83)	< 0.001
Obese (BMI ≥ 27)	213/4583 (4.6)	0.61 (0.52 to 0.71)	< 0.001

BMI: body mass index; HR: hazard ratio; CI: confidence interval. Multivariable model: adjustment for age, sex, history of hypertension, history of diabetes mellitus, history of dyslipidemia, serum albumin, fasting glucose, and total cholesterol.

Unit of BMI = kg/m^2^.

### Subjects in the underweight group had the highest decrease in BMD T-score compared to the other groups

We further examined differences between BMI groups and changes in BMD T-score. The ΔBMD T-scores were − 0.463 ± 1.0874, − 0.338 ± 0.9831, − 0.286 ± 0.9249, and − 0.257 ± 1.0133 in the underweight, normal weight, overweight, and obese groups, respectively. The underweight group had the highest decrease in BMD T-score compared to the other groups, and this decrease was significant when compared with the normal weight group (p value = 0.008) (Table 5).

**Table 5:** The values of BMD T score (baseline), BMD T score (follow-up) and ΔBMD T score among different BMI groups (n = 24,507).

BMI groups	BMD T score (baseline)	BMD T score (follow-up)	ΔT score	p value (ΔT score)
Underweight (BMI < 18.5)	− 0.311 ± 1.5124	− 0.774 ± 1.4628	− 0.463 ± 1.0874	0.008
Normal weight (18.5 ≤ BMI < 24)	− 0.251 ± 1.472	− 0.589 ± 1.4733	− 0.338 ± 0.9831	–
Overweight (24 ≤ BMI < 27)	− 0.264 ± 1.4018	− 0.551 ± 1.4304	− 0.286 ± 0.9249	0.003
Obese (BMI ≥ 27)	− 0.113 ± 1.4823	− 0.370 ± 1.5180	− 0.257 ± 1.0133	< 0.001

BMD: bone mineral density. Unit of BMI = kg/m^2^.

## Discussion

In this large-scale, population-based study, the underweight individuals exhibited an increased risk of osteoporosis after adjusting for confounding factors compared to the other weight groups. Moreover, they had the most significant decrease in BMD T-score compared to the other groups over a mean follow-up period of 47 months. To the best of our knowledge, this is the largest study to demonstrate underweight status as an independent risk factor for the development of osteoporosis.

Numerous studies have demonstrated a positive relationship between BMI and BMD. For example, Walsh and colleagues reported a significant correlation between BMI and BMD, and proposed that potential mechanisms may include increased loading and heightened aromatase activity^32^. Another study conducted in the US revealed that each unit increase in BMI was linked to a 0.0082 g/cm^2^ increase in BMD (p value < 0.001)^33^. In addition, Felson et al. demonstrated the protective influence of higher body weight on BMD levels across various sites, especially in weight-bearing bones^34^. Similarly, an Asian study identified positive correlations between body weight, BMI, height, and BMD at different anatomical locations (p value < 0.05)^35^. In line with these findings, we found a robust association between BMI and osteoporosis. Notably, most previous studies have been cross-sectional, whereas we conducted a large longitudinal study and provided evidence that being underweight was an independent risk factor for developing osteoporosis.

While being underweight has been correlated with a reduced risk of cardiovascular disease, a lower weight status may be harmful to health due to suboptimal nutrition and reduced muscle mass and strength^36^. Moreover, decreased mechanical loading and muscle stress on bones can potentially lead to lower peak bone mass and increased bone loss^37^. Our findings support these studies, and demonstrated a higher incidence of osteoporosis in the underweight group compared to the normal weight group. Consequently, maintaining a healthy BMI may be an important and modifiable factor in osteoporosis prevention. A study by Lee et al. reinforces this idea, in which they identified an optimal BMI range of 23.0 to 24.9 kg/m^2^ to minimize the risk of osteoporosis^38^. Beyond this range, the risk of osteoporosis decreases. Conversely, a BMI below this range may increase the risk of osteoporosis.

In the present study, underweight was not only a risk factor for the development of osteoporosis, but it was also related to a significant reduction in BMD T score (ΔBMD T-score of − 0.463 ± 1.0874). Meyer et al. investigated how weight fluctuations over three decades impacted the risk of osteoporosis, and they found that individuals who lost < 5% of their body weight had a 6.2% prevalence of osteoporosis, rising to 14.1% for a 5–10% loss and 15.1% for a > 10% loss^13^. In contrast, 2.6% of those with a 5–10% weight gain had osteoporosis, compared to 0.6% of those with a > 10% gain^13^. The authors linked a low BMI to a higher risk of osteoporosis, emphasizing the pivotal role of stable weight in maintaining bone health. Their findings also underscored the impact of weight fluctuations (> 0.25 kg/year) on BMD^13^. Rapid weight loss, especially from dieting can irreversibly decrease BMD^39^, highlighting the necessity of gradual, healthy weight management for long-term bone health.

Several modifiable factors play a crucial role in promoting bone health^40–44^. According to Christianson et al.^40^ and other studies^41,42^, regular weight-bearing exercise and a balanced diet with adequate calcium, vitamin D, and protein intake are essential recommendations. In addition, other studies have shown that daily tea consumption positively influences BMD in osteoporotic women, while excessive salt and coffee consumption have negative effects on BMD^43^. Regular weight-bearing exercise can not only promote bone health but also enhance balance and motor strength, reducing the risk of falls^40^. Soltani et al. found that weight loss was associated with reduced BMD at the hip, with a more significant impact than on the spine. Calorie restriction and a combination of calorie restriction and exercise have been associated with decreased hip BMD, whereas exercise training without dietary restriction has been associated with increased hip BMD^44^. Moreover, critical modifiable lifestyle factors associated with bone health and decrease fracture risk include avoiding smoking, maintaining a healthy body weight (especially BMI ≥ 20 kg/m^2^), limiting alcohol intake, and minimizing the risk of falls at home^40^.

Two potential mechanisms have been proposed to explain how body mass affects osteoporosis. The first mechanism involves mechanical loading, where additional weight imposes higher static mechanical stress on bones^14^. This stress can then trigger adaptive responses, leading to changes in bone quality and structure^14^. Heavy individuals tend to attain higher peak BMD in early adulthood, which exerts a greater load on weight-bearing joints and results in higher BMD, reducing the likelihood of osteoporosis in old age^14^. The second mechanism involves the physiological function of adipose tissue, which influences bone through an endocrine pathway^45–47^. Adipose tissue impacts bone metabolism by metabolizing sex steroids, indirectly protecting against bone loss. Adipose tissue expresses and secretes adipocytokines such as leptin and adiponectin^45–47^. Leptin stimulates osteoblast proliferation, mineralization, collagen synthesis, and inhibits bone resorption, while adiponectin promotes excessive bone resorption associated with bone loss, negatively affecting BMD, particularly in postmenopausal women^45,46^. Current evidence indicates that leptin positively affects BMI, while adiponectin is negatively associated with BMD, making it a relevant adipokine negatively linked to postmenopausal osteoporosis^47^.

The strengths of this study include that it is the first large-scale, cross-sectional, and longitudinal study to explore the interconnection between BMI and osteoporosis among an Asian Han population. Utilizing a nationally representative cohort of over 120,000 Taiwanese men and women greatly enhanced the statistical power of our results. Not only did we establish a clear link between BMI and osteoporosis prevalence, but we also identified the incidence of newly developed osteoporosis. Our findings offer invaluable insights for healthcare professionals, empowering them to engage in informed discussions with patients about the impact of BMI status on bone health. Nonetheless, our study also has limitations that warrant acknowledgment. Firstly, we used calcaneal QUS to assess BMD, while previous studies have utilized dual energy X-ray absorptiometry (DXA)-derived BMD. The choice of measurement technique stems from the advantages of QUS, which is commonly used for peripheral bones such as the calcaneus. The calcaneus is primarily composed of cancellous bone with minimal soft tissues and a large measurable plane, making it ideal for QUS assessments^48^. Discrepancies between QUS and DXA measurements arise from the fundamental differences in technology and the measurement site. The calcaneus, with a lower cortical bone proportion, is subject to distinct loading mechanisms compared to the proximal femur^49^. Despite this, numerous studies have validated that calcaneal QUS is a reliable tool for assessing BMD and shown its efficiency in diagnosing osteoporosis^48^. Secondly, we did not monitor weight changes in the participants, limiting our ability to substantiate the impact of weight gain on bone loss. In addition, we did not explore correlations between BMD and other health indicators such as fat mass, lean mass, fat distribution, peripheral and visceral fat tissue, and waist circumference, all of which may influence BMD. Thirdly, although we adjusted for several covariates, some crucial confounding factors were not considered, including the use of medications affecting bone metabolism, specific hormone therapies, nutritional supplements, serum calcium levels, and dietary patterns. Other factors such as prior fractures, surgical history, and other disorders, which could have significantly impacted our correlation analyses, were also omitted. Furthermore, due to the absence of data on prior fractures, we could not assess the relationship between osteoporotic fracture risk and BMI.

## Conclusion

Our results suggest that BMI is associated with both the prevalence and the incidence of osteoporosis. In addition, underweight is an independent risk factor for developing osteoporosis. These findings highlight the importance of maintaining normal weight for optimal bone health.

### Supplementary Information

Supplementary Tables.

## Data Availability

The data underlying this study are from the Taiwan Biobank. Due to restrictions placed on the data by the Personal Information Protection Act of Taiwan, the minimal data set cannot be made publicly available. Data may be available upon request to interested researchers. Please send data requests to Szu-Chia Chen, Division of Nephrology, Department of Internal Medicine, Kaohsiung Medical University Hospital, Kaohsiung Medical University.
