# Supplementary material for: The association between body mass index and osteoporosis in a Taiwanese population: a cross-sectional and longitudinal study
Source: Sci Rep. 2024 Apr 12;14:8509. doi: 10.1038/s41598-024-59159-4 (PMC11009266; doi:10.1038/s41598-024-59159-4)
Supplement: Supplementary file 1 — Supplementary Tables. [file 41598_2024_59159_MOESM1_ESM.docx]

Supplementary Material

**Supplementary Table S1. Univariable associations between clinical factors and osteoporosis in cross-sectional analysis (n = 119,009)**

| Characteristics | OR (95% CI) | *p* value |
| --- | --- | --- |
| Age (per 1 year) | 1.08 (1.08 to 1.08) | <0.001 |
| Sex (female vs. male) | 0.78 (0.75 to 0.82) | <0.001 |
| Smoking (ever vs. never) | 1.16 (1.11 to 1.21) | <0.001 |
| Alcohol status (ever vs. never) | 1.19 (1.12 to 1.28) | <0.001 |
| Hypertension (yes vs. no) | 1.54 (1.46 to 1.63) | <0.001 |
| Diabetes (yes vs. no) | 1.44 (1.32 to 1.56) | <0.001 |
| Dyslipidemia (yes vs. no) | 1.44 (1.34 to 1.54) | <0.001 |
| Hemoglobin (per 1 g/dl) | 1.02 (1.01 to 1.03) | 0.002 |
| Albumin (per 1 g/dl) | 0.65 (0.60 to 0.71) | <0.001 |
| Fasting Glucose (per 1 mg/dl) | 1.01 (1.00 to 1.01) | <0.001 |
| Total cholesterol (per 1 mg/dl) | 1.00 (1.00 to 1.00) | <0.001 |
| Triglyceride (per 1 mg/dl) | 1.00 (1.00 to 1.00) | <0.001 |
| Uric acid (per 1 mg/dL) | 1.01 (0.99 to 1.03) | 0.155 |
| eGFR (per 1 ml/min/1.73 m^2^) | 0.99 (0.99 to 1.00) | <0.001 |
| BMI groups (Underweight vs. Normal weight) | 1.64 (1.49 to 1.80) | <0.001 |
| BMI groups (Overweight vs. Normal weight) | 0.88 (0.84 to 0.92) | <0.001 |
| BMI groups (Obese vs. Normal weight) | 0.72 (0.68 to 0.76) | <0.001 |

BMI = body mass index; OR = odds ratio; CI = Confidence interval; eGFR = Estimated glomerular filtration rate.

**Supplementary Table S2. Univariable and multivariable binary logistic analysis for the prevalence of osteoporosis in subgroup analyses (n = 119,009)**

| Characteristics | Unadjusted odds ratio (95% CI) | *p* value | Adjusted odds ratio (95% CI) | *p* value |
| --- | --- | --- | --- | --- |
| **Gender, female** | | | | |
| Underweight (BMI < 18.5) | 1.52 (1.36 to 1.69) | <0.001 | 2.09 (1.85 to 2.36) | <0.001 |
| Normal weight (18.5≦BMI<24) | 1.00 (Reference) | - | 1.00 (Reference) | - |
| Overweight (24≦BMI<27) | 0.86 (0.80 to 0.92) | <0.001 | 0.72 (0.67 to 0.77) | <0.001 |
| Obese (BMI≧27) | 0.67 (0.61 to 0.72) | <0.001 | 0.63 (0.57 to 0.69) | <0.001 |
| **Gender, male** | | | | |
| Underweight (BMI < 18.5) | 2.82 (2.32 to 3.43) | <0.001 | 2.94 (2.40 to 3.61) | <0.001 |
| Normal weight (18.5≦BMI<24) | 1.00 (Reference) | - | 1.00 (Reference) | - |
| Overweight (24≦BMI<27) | 0.78 (0.72 to 0.84) | <0.001 | 0.78 (0.72 to 0.85) | <0.001 |
| Obese (BMI≧27) | 0.66 (0.60 to 0.71) | <0.001 | 0.73 (0.66 to 0.80) | <0.001 |
| **Age > 65 years old** |  |  |  |  |
| Underweight (BMI < 18.5) | 2.62 (1.98 to 3.47) | <0.001 | 2.27 (1.71 to 3.03) | <0.001 |
| Normal weight (18.5≦BMI<24) | 1.00 (Reference) | - | 1.00 (Reference) | - |
| Overweight (24≦BMI<27) | 0.77 (0.68 to 0.87) | <0.001 | 0.86 (0.76 to 0.98) | 0.021 |
| Obese (BMI≧27) | 0.61 (0.52 to 0.71) | <0.001 | 0.69 (0.58 to 0.81) | <0.001 |
| **Age ≦ 65 years old** |  |  |  |  |
| Underweight (BMI < 18.5) | 1.60 (1.45 to 1.77) | <0.001 | 2.21 (1.98 to 2.46) | <0.001 |
| Normal weight (18.5≦BMI<24) | 1.00 (Reference) | - | 1.00 (Reference) | - |
| Overweight (24≦BMI<27) | 0.87 (0.82 to 0.92) | <0.001 | 0.72 (0.68 to 0.76) | <0.001 |
| Obese (BMI≧27) | 0.73 (0.69 to 0.78) | <0.001 | 0.68 (0.64 to 0.73) | <0.001 |

CI = Confidence interval.

Multivariable model: adjustment for age, sex, smoking, drinking, history of hypertension, history of diabetes mellitus, history of dyslipidemia, serum albumin, fasting glucose, total cholesterol, triglyceride, albumin, uric acid and estimated glomerular filtration rate.

Unit of BMI = kg/m^2^.

**Supplementary Table S3. Univariable associations between clinical factors and osteoporosis in longitudinal analysis (n = 24,507)**

| Characteristics | HR (95% CI) | *p* value |
| --- | --- | --- |
| Age (per 1 year) | 1.07 (1.07 to 1.08) | <0.001 |
| Sex (female vs. male) | 1.12 (1.01 to 1.25) | 0.037 |
| Smoking (ever vs. never) | 0.97 (0.86 to 1.09) | 0.602 |
| Alcohol status (ever vs. never) | 1.08 (0.90 to 1.29) | 0.399 |
| Hypertension (yes vs. no) | 1.42 (1.24 to 1.64) | <0.001 |
| Diabetes (yes vs. no) | 1.32 (1.07 to 1.63) | 0.009 |
| Dyslipidemia (yes vs. no) | 1.25 (1.04 to 1.50) | 0.019 |
| Hemoglobin (per 1 g/dl) | 0.99 (0.96 to 1.02) | 0.592 |
| Albumin (per 1 g/dl) | 0.58 (0.47 to 0.71) | <0.001 |
| Fasting Glucose (per 1 mg/dl) | 1.00 (1.00 to 1.01) | 0.006 |
| Total cholesterol (per 1 mg/dl) | 1.01 (1.00 to 1.01) | <0.001 |
| Triglyceride (per 1 mg/dl) | 1.00 (1.00 to 1.00) | 0.747 |
| Uric acid (per 1 mg/dL) | 0.97 (0.94 to 1.01) | 0.092 |
| eGFR (per 1 ml/min/1.73 m^2^) | 1.00 (1.00 to 1.00) | 0.094 |
| BMI groups (Underweight vs. Normal weight) | 1.33 (1.02 to 1.72) | 0.032 |
| BMI groups (Overweight vs. Normal weight) | 0.83 (0.74 to 0.94) | 0.002 |
| BMI groups (Obese vs. Normal weight) | 0.65 (0.56 to 0.75) | <0.001 |

HR = hazard ratio; CI = Confidence interval; eGFR = Estimated glomerular filtration rate.
